# Association of four imprinting disorders and ART

**DOI:** 10.1186/s13148-019-0623-3

**Published:** 2019-02-07

**Authors:** Hiromitsu Hattori, Hitoshi Hiura, Akane Kitamura, Naoko Miyauchi, Norio Kobayashi, Souta Takahashi, Hiroaki Okae, Koichi Kyono, Masayo Kagami, Tsutomu Ogata, Takahiro Arima

**Affiliations:** 1 Department of Informative Genetics, Environment and Genome Research Center, Tohoku University Graduate School of Medicine, 2-1 Seiryo-cho, Aoba-ku, Sendai, 980-8575 Japan; 2 Kyono ART Clinic, 1-1-1, Honcho, Aoba-ku, Sendai, 980-0014 Japan; 3 Department of Molecular Endocrinology, National Research Institute for Child Health and Development, 2-10-1 Ohkura, Setagaya-ku, Tokyo, 157-8535 Japan; 4 Department of Pediatrics, Hamamatsu University School of Medicine, 1-20-1, Handayama, Higashi-ku, Hamamatsu, 431-3192 Japan

**Keywords:** Imprinting disorders, Assisted reproductive technologies (ART), Silver-Russell syndrome (SRS), Nationwide epidemiological study, DNA methylome, DNA methylation variations (DMVs)

## Abstract

**Background:**

Human-assisted reproductive technologies (ART) are a widely accepted treatment for infertile couples. At the same time, many studies have suggested the correlation between ART and increased incidences of normally rare imprinting disorders such as Beckwith-Wiedemann syndrome (BWS), Angelman syndrome (AS), Prader-Willi syndrome (PWS), and Silver-Russell syndrome (SRS). Major methylation dynamics take place during cell development and the preimplantation stages of embryonic development. ART may prevent the proper erasure, establishment, and maintenance of DNA methylation. However, the causes and ART risk factors for these disorders are not well understood.

**Results:**

A nationwide epidemiological study in Japan in 2015 in which 2777 pediatrics departments were contacted and a total of 931 patients with imprinting disorders including 117 BWS, 227 AS, 520 PWS, and 67 SRS patients, were recruited. We found 4.46- and 8.91-fold increased frequencies of BWS and SRS associated with ART, respectively. Most of these patients were conceived via in vitro fertilization (IVF) and intracytoplasmic sperm injection (ICSI), and showed aberrant imprinted DNA methylation. We also found that ART-conceived SRS (ART-SRS) patients had incomplete and more widespread DNA methylation variations than spontaneously conceived SRS patients, especially in sperm-specific methylated regions using reduced representation bisulfite sequencing to compare DNA methylomes. In addition, we found that the ART patients with one of three imprinting disorders, PWS, AS, and SRS, displayed additional minor phenotypes and lack of the phenotypes. The frequency of ART-conceived Prader-Willi syndrome (ART-PWS) was 3.44-fold higher than anticipated. When maternal age was 37 years or less, the rate of DNA methylation errors in ART-PWS patients was significantly increased compared with spontaneously conceived PWS patients.

**Conclusions:**

We reconfirmed the association between ART and imprinting disorders. In addition, we found unique methylation patterns in ART-SRS patients, therefore, concluded that the imprinting disorders related to ART might tend to take place just after fertilization at a time when the epigenome is most vulnerable and might be affected by the techniques of manipulation used for IVF or ICSI and the culture medium of the fertilized egg.

**Electronic supplementary material:**

The online version of this article (10.1186/s13148-019-0623-3) contains supplementary material, which is available to authorized users.

## Background

Human-assisted reproductive technologies (ART) are becoming increasingly common due to late marriage and improvements in medical technology in developed countries, including Japan [1]. The majority of studies published previously have suggested that babies conceived after ART have increased incidences of normally rare imprinting disorders such as Beckwith-Wiedemann syndrome (BWS), Angelman syndrome (AS), Prader-Willi syndrome (PWS), and Silver-Russell syndrome (SRS) [2–7]. On the other hand, some studies found no relation between ART and imprinting disorders [8, 9]. Imprinting disorders are caused by genetic defects or epigenetic mutations (DNA methylation); i.e., aberrant DNA methylation of differentially methylated regions (DMRs) that regulate allele-specific expression of imprinted genes [10]. The relationship between ART and aberrant genomic imprinting is still unclear. However, many studies have suggested that ovarian stimulation, culture media used for gametes and early embryos, in vitro fertilization (IVF) and intracytoplasmic sperm injection (ICSI) manipulations, and the freezing and thawing of embryos may prevent the proper establishment and maintenance of genomic imprinting and cause imprinting disorders [11, 12].

In 2009, we performed a nationwide epidemiological study in Japan to determine the frequencies of four imprinting disorders (BWS, AS, PWS, and SRS) and found that the frequencies of ART-conceived BWS (ART-BWS) and ART-SRS patients were nearly tenfold higher than anticipated [13]. In addition, the majority of ART-SRS and ART-BWS patients showed aberrant methylation not only in domains responsible for imprinting disorders but also in additional multiple imprinted loci. Other reports have also described multi-locus imprinting disturbances after ART, supporting our hypothesis [14–16]. Furthermore, in addition to aberrant DNA methylation patterns at multiple imprinted loci, there are also mosaic methylation errors. We assumed that the imprinting errors occurred after fertilization rather than in the gametes. However, it is unknown who is predisposed to the imprinting disorders and which factor(s) of ART cause them.

In this study, we conducted another Japanese nationwide epidemiological study of the imprinting disorders in 2015 and reconfirmed the tight association between ART and imprinting disorders. Next, we studied new patients with SRS and found significant differences in the genome-wide DNA methylation between the ART patients and spontaneously conceived (Sp) patients.

## Results

### Relationship between the imprinting disorders and ART

We conducted a Japanese nationwide epidemiological study of four imprinting disorders to determine associations with ART. We contacted a total of 2777 pediatric departments of all hospitals that were identified based on a hospital lists. In all, 1957 departments (70.5%) responded to the first-stage survey questionnaire. We inquired about detailed clinical information and examined the relationship with ART using a second-stage survey ensuring the exclusion of duplicates and a total of 931 patients with imprinting disorders were recruited. The number, age, and gender distributions of patients are given in Additional file 1. The numbers of patients with the four imprinting disorders slightly increased year by year, especially for BWS and SRS. However, there were no differences in the gender ratios of the imprinting disorders. We ascertained the frequencies of ART-BWS, ART-AS, ART-PWS, and ART-SRS via a questionnaire sent to doctors (Table 1). The observed/expected ratios for four ART-imprinting disorders were calculated by considering the live birth rate after ART (1.34%) from 1985 through 2015 in Japan (http://plaza.umin.ac.jp/~jsog-art/). For BWS and SRS, the number of ART patients were 4.46- and 8.91-fold higher than anticipated, accounting for 6.0% (7/117) of BWS and 11.9% (8/67) of SRS patients, respectively. These proportions were similar to those we found in 2009 [13]. The number of ART-PWS patients was 3.44-fold higher than anticipated, but no increase in ART-AS patients was observed compared with that anticipated (1.8%, 4/227).

**Table 1:** Frequency of ART in children with four imprinting disorders and observed/expected ratios of ART in the same groups

	The percentage of patients after ART/total: % (*n*) (2015)	Observed/expected ratios of ART
BWS	6.0% (7/117)	4.46
AS	1.8% (4/227)	1.32
PWS	4.6% (24/520)	3.44
SRS	11.9% (8/67)	8.91

Results of a nationwide epidemiological investigation of four imprinting disorders in Japan, under the governance of the Ministry of Health, Labor and Welfare of the Japanese government. Whether the patient was born after ART treatment was confirmed in a questionnaire. The number of patients was expected by multiplying the total number of disease patients by the live birth rate after ART (1.34%) from 1985 through 2015 in Japan (http://plaza.umin.ac.jp/~jsog-art/)

In the second questionnaire, we examined the results of genetic and DNA methylation tests (Table 2 and Additional file 2). In both ART-BWS and ART-SRS patients, DNA methylation error rates were 100% (4/4 and 5/5, respectively). Additionally, that in ART-BWS was significantly higher than in spontaneously conceived BWS (Sp-BWS) patients (*p* = 0.027). Nearly one-half of ART-BWS (42.9%, 3/7) and the majority of ART-SRS (75%, 6/8) patients received IVF or ICSI treatment (Additional file 3). The rate of maternal uniparental disomy of chromosome 15 (UPD(15)mat) in ART-PWS patients was significantly increased compared with that of Sp-PWS patients (*p* = 0.001). Next, we classified PWS patients into two categories based on maternal ages ≤ 37 years and ≥ 38 years and compared ART patients with Sp patients since the aneuploidy rate in the human embryo dramatically increases when the maternal age ≥ 38 years [17]. The rate of DNA methylation errors in ART-PWS patients was significantly increased compared with that in Sp-PWS patients for maternal age ≤ 37 years, whereas the rate of UPD(15)mat in ART-PWS patients was significantly increased compared with that in Sp-PWS patients with maternal age ≥ 38 years (*p* = 0.021, Table 3 and Additional file 4). There was no correlation between maternal age and UPD in BWS, AS, and SRS patients (Additional file 5).

**Table 2:** Pathogeneses of four imprinting disorders

		UPD	Gene mutations	Deletions	DNA methylation errors
BWS (*n* = 43)	ART (*n* = 4)	–	–	–	100% (4/4)*
	Sp (*n* = 39)	46.2% (18/39)	2.6% (1/39)	7.7% (3/39)	43.6% (17/39)
AS (*n* = 147)	ART (*n* = 4)	–	–	100% (4/4)	–
	Sp (*n* = 143)	2.1% (3/143)	3.5% (5/143)	92.3% (132/143)	2.1% (3/143)
PWS (*n* = 366)	ART (*n* = 21)	42.9% (9/21)*	–	28.6% (6/21)	28.6% (6/21)
	Sp (*n* = 345)	13.3% (46/345)	–	73.6% (254/345)*	13.0% (45/345)
SRS (*n* = 22)	ART (*n* = 5)	–	–	–	100% (5/5)
	Sp (*n* = 17)	23.5% (4/17)	–	–	76.5% (13/17)

The numbers and percentages of patients with chromosomal abnormalities, gene mutations, and methylation abnormalities were obtained from a questionnaire. An asterisk indicates a significant difference between ART-patients and Sp-patients (*p* < 0.05). For BWS, UPD, and gene indicate paternally uniparental disomy of chromosome 11 and *CDKN1C*, and methylation errors include both gain of methylation at *H19*/*IGF2* IG-DMR and loss of methylation at *KCNQ1OT1*:TSS-DMR, respectively. For AS, UPD, gene, and methylation error indicate paternally uniparental disomy of chromosome 15, *UBE3A* and loss of methylation at *SNRPN*-DMR, respectively. For PWS, UPD, and methylation error indicate maternally uniparental disomy of chromosome 15 and gain of methylation at *SNRPN*-DMR, respectively. For SRS, UPD, and methylation error indicate maternally uniparental disomy of chromosome 7 and loss of methylation at *H19/IGF2* IG-DMR, respectively. *UPD* uniparental disomy

**Table 3:** Frequency of different pathogeneses in ART patients and Sp patients with PWS stratified according to maternal age

	Maternal age ≤ 37 years			Maternal age ≥ 38 years		
	ART patients (*n* = 12)	Sp patients (*n* = 215)	*P* value	ART patients (*n* = 9)	Sp patients (*n* = 44)	*P* value
UPD(15)mat	16.7% (2/12)	9.8% (21/215)	0.348	77.8% (7/9)	31.8% (14/44)	0.021
Microdeletion at chromosome15q11.5 region	50.0% (6/12)	79.5% (171/215)	0.027	0% (0/9)	36.4% (16/44)	0.044
DNA methylation error	33.3% (4/12)	10.7% (23/215)	0.041	22.2% (2/9)	31.8% (14/44)	0.706

All data were obtained from the questionnaire. DNA methylation error indicates gain of methylation at *SNRPN*-DMR. UPD(15)mat, maternal uniparental disomy of chromosome 15

### Phenotypic differences in patients conceived with ART

To investigate whether disease phenotypes in patients could be altered by ART, we compared the clinical features in detail. There were no major differences overall between ART patients and Sp patients for the four diseases, but there were some significant phenotypic differences (Table 4). For ART-SRS patients, the frequency of heart malformation was significantly higher (25.0%, 2/8; 3.4%, 2/59, *p* = 0.036) than in Sp-SRS patients. In addition, the frequency of mental retardation in ART-SRS was higher (62.5%, 5/8; 32.2%, 19/59, *p* = 0.124) than in Sp-SRS patients. Tumorigenesis and diabetes occurred in only one ART-SRS patient. For ART-AS patients, the frequencies of hyposomnia and ataxic gait were significantly higher (100%, 4/4; 46.2%, 103/223, *p* = 0.048) and lower (0%, 0/4; 60.1%, 134/223, *p* = 0.027), respectively, than in Sp-AS patients. For ART-PWS patients, the frequencies of dyschromatosis (91.7%, 22/24; 69.0%, 342/496, *p* = 0.020) and acromicria (87.5%, 21/24; 58.3%, 289/496, *p* = 0.005) were significantly higher and the frequency of obesity was lower (14.3%, 2/14; 49.4%, 205/415, *p* = 0.012) than in Sp-PWS patients. For BWS, there were some phenotypical differences between ART-BWS and Sp-BWS patients, but these were not significant.

**Table 4:** Clinical phenotypic characterization of patients with ART

	ART (*n* = 7)	Sp (*n* = 110)	*P* value
a. BWS			
Macroglossia (%)	100	82.7	0.597
Earlobe creases (%)	57.1	43.6	0.698
Umbilical hernia (%)	57.1	44.5	0.700
Hemihypertrophy (%)	28.6	34.5	1.000
Exomphalos (%)	42.9	22.7	0.356
Exophthalmos (%)	28.6	19.1	0.622
Hepatomegaly (%)	42.9	16.4	0.108
Nephromegaly (%)	14.3	16.4	1.000
Ocular hypertelorism (%)	14.3	13.6	1.000
Urinary malformation (%)	0	10.9	1.000
Cryptorchism (%)	28.6	10.0	0.174
Occlusal interference (%)	14.3	10.9	0.572
Pancreatic islet hyperplasia (%)	0	8.2	1.000
Adrenomegaly (%)	0	4.5	1.000
Splenomegaly (%)	0	3.6	1.000
	ART (*n* = 4)	Sp (*n* = 223)	*P* value
b. AS			
Mental retardation (%)	100	98.7	1.000
Epilepsy (%)	100	65.9	0.304
Dysphasia (%)	100	89.2	1.000
Hyposomnia (%)	100	46.2	0.048
Dyschromatosis (%)	75.0	73.5	1.000
Prognathism (%)	50.0	50.7	1.000
Convulsions (%)	50.0	35.0	0.615
Microcephaly (%)	50.0	25.6	0.204
Ictal laughter (%)	25.0	57.0	0.320
Ataxic gait (%)	0	60.1	0.027
Heart malformation (%)	0	2.7	1.000
	ART (*n* = 24)	Sp (*n* = 496)	*P* value
c. PWS			
Hypotonia (%)	100	86.9	0.059
Dyschromatosis (%)	91.7	69.0	0.020
Feeding difficulties (%)	91.7	79.9	0.194
Almond-shaped eyes (%)	87.5	73.6	0.156
Acromicria (%)	87.5	58.3	0.005
Mental retardation (%)	79.2	86.1	0.365
Short stature (%)	62.5	64.4	0.831
Triangular mouth (%)	66.7	61.6	0.673
Cryptorchism (%)	58.3	38.6	0.085
Hypogonadism (%)	50.0	37.4	0.281
Bulimia (%)	35.7	52.3	0.281
Obesity (%)	14.3	49.4	0.012
Diabetes (%)	8.3	12.7	0.755
Gastrointestinal injury (%)	8.3	2.8	0.165
Cardiac failure (%)	4.2	2.8	0.512
	ART (*n* = 8)	Sp (*n* = 59)	*P* value
d. SRS			
Short stature (%)	100	94.9	1.000
Failure to thrive (%)	100	94.9	1.000
Triangular shaped face (%)	87.5	78.0	1.000
Body asymmetry (%)	87.5	57.6	0.138
Clinodactyly of the fifth fingers (%)	62.5	47.5	0.476
Mental retardation (%)	62.5	32.2	0.124
Sweating (%)	25.0	15.3	0.609
Heart malformation (%)	25.0	3.4	0.036
Hypoglycemia (%)	0	10.2	1.000
Gastrointestinal injury (%)	0	6.8	1.000
Difficulty in hearing (%)	0	3.4	1.000
Ptosis (%)	0	5.1	1.000
Renal hypoplasia (%)	0	1.7	1.000
Tumorigenesis (%)	12.5	0	0.119
Diabetes (%)	12.5	0	0.119
Hypertension (%)	0	1.7	1.000

The percentages of patients presenting each clinical phenotypic in four ART- and Sp-imprinting disorders: (a) BWS, (b) AS, (c) PWS, and (d) SRS. The frequencies of bulimia and obesity were calculated for PWS patients over 3 years old. The total numbers of ART-PWS and Sp-PWS patients over 3 years old were 14 and 415, respectively

### Genome-wide methylation in ART-SRS patients

To investigate the genome-wide DNA methylation changes in ART-SRS patients, we performed reduced representation bisulfite sequencing (RRBS) to produce DNA methylomes derived from the peripheral blood of five ART-SRS patients with *H19*/*IGF2* intergenic (IG)-DMR hypomethylation, five Sp-SRS patients with *H19*/*IGF2* IG-DMR hypomethylation, and ten Sp-normal children (control) who were randomly selected. We obtained an average of 3,242,341 CpG cytosines per sample and analyzed 2,069,685 CpG cytosines covered in the autosomes of all samples (Additional file 6). Global evaluations of the DNA methylation levels of all CpG cytosines in the genome among individuals of the three groups were very similar (*R* > 0.975), and the correlation coefficients between individuals within the ART-SRS and Sp-SRS groups were greater than between individuals within the control group (Additional file 7). Among the mean methylation levels of the various genomic regions, the most methylated was in the ART-SRS group, followed in order by the Sp-SRS and control groups. The mean methylation levels of the gene bodies, exons, introns, intergenic regions, CpG island (CGI) shores, short interspersed nuclear elements (SINEs), long interspersed nuclear elements (LINEs), long terminal repeats (LTRs), and DNA repeats in the ART-SRS and Sp-SRS groups were significantly increased compared to the control group. In addition, the mean methylation levels of the CGI shelves and simple repeats in the ART-SRS group were significantly increased compared to the control group (Additional file 8).

### Characterization of DMVs in ART-SRS patients

**Fig. 1:**
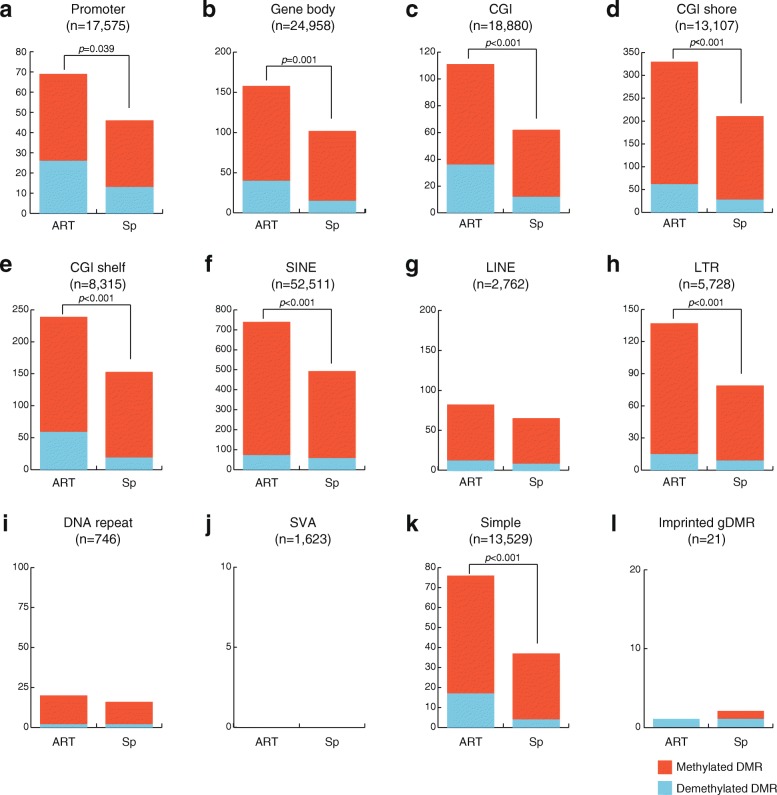
Comparison of the numbers of DMVs in ART-SRS and Sp-SRS groups. **a** Promoter. **b** Gene body. **c** CGI. **d** CGI shore. **e** CGI shelf. **f** SINE. **g** LINE. **h** LTR. **i** DNA repeat. **j** SVA. **k** Simple repeat. **l** Imprinted gDMR. Red and blue bars indicate the numbers of methylated DMVs and demethylated DMVs, respectively. *DMV* DNA methylation variation, *CGI* CpG island, *SINE* short interspersed nuclear element, *LINE* long interspersed nuclear element, *LTR* long terminal repeat element, *SVA* SINE-VNTR-Alu, *gDMR* germline differentially methylated region

We annotated genomic regions and then identified the DNA methylation variation (DMVs) showing absolute methylation changes ≥ 7.5% and statistically significant differences (false discovery rate (FDR) < 0.05) in the ART-SRS and Sp-SRS groups compared with the control group (Fig. 1). The numbers of DMVs in the ART-SRS group were significantly increased compared with those in the Sp-SRS group in the promoter, gene body, CGI, CGI shore, CGI shelf, SINE, LTR, and simple repeat regions. In addition, the number of methylated DMVs was larger than that of demethylated DMVs in all regions. On the other hand, we found that there were no more DMVs in the ART-SRS and Sp-SRS groups than in the control group for SINE-VNTR-Alu (SVA).

**Fig. 2:**
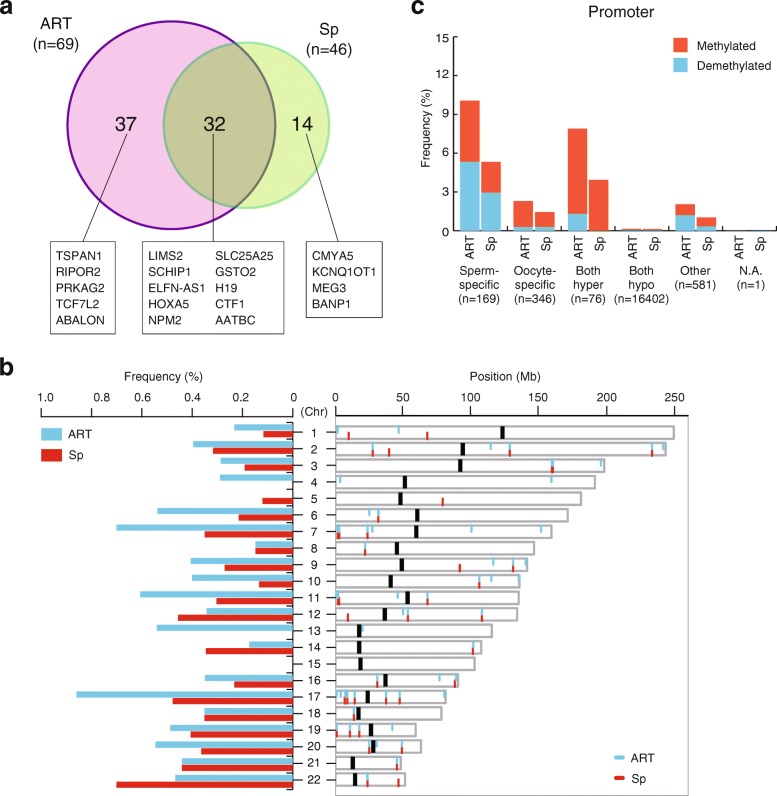
Promoters with DMVs in SRS patients. **a** A Venn diagram showing 83 promoters with DMVs in SRS patients. **b** Autosomal chromosome frequency (left) and distribution (right) of the promoters with DMVs. Blue bars represent DMVs in ART-SRS patients and red bars represent DMVs in Sp-SRS patients. N.A. indicates that data was not available. **c** DMVs of promoters in ART-SRS and Sp-SRS patients classified based on methylation of gametes. Sperm-specific methylated regions were ≥ 80% methylated in sperm and ≤ 20% methylated in oocytes, oocyte-specific methylated regions were ≤ 20% methylated in sperm and ≥ 80% methylated in oocytes, both hypermethylated regions were ≥ 80% methylated in both sperm and oocytes, both hypomethylated regions were ≤ 20% methylated in both sperm and oocytes according to our previously reported data [18]

To investigate genes related to the characteristics of SRS, we next focused on promoter regions. A total of 83 promoters (corresponding to 79 genes) showed DMVs; 37 promoters (36 genes) with DMVs only in the ART-SRS group, 14 promoters (13 genes) with DMVs only in the Sp-SRS group, and 32 promoters (30 genes) with DMVs in both the ART-SRS and Sp-SRS groups (Fig. 2a and Additional file 9). These promoters with DMVs were distributed in almost all autosomes (Fig. 2b). We used the Database for Annotation, Visualization and Integrated Discovery (DAVID) to interpret the biological meanings of lists of ART-SRS-specific, Sp-SRS-specific, and all genes with DMVs but there were no significant gene ontology (GO) terms. However, some genes with DMVs might be associated with clinical phenotypes of SRS; therefore, we describe these genes below (see “Discussion” section).

### Germline-specific methylation patterns of identified DMVs in ART-SRS patients

To investigate the characteristics of DMVs identified in ART-SRS patients, we classified the DMVs into four categories: sperm-specific methylated regions ≥ 80% methylated in sperm and ≤ 20% methylated in oocytes, oocyte-specific methylated regions ≤ 20% methylated in sperm and ≥ 80% methylated in oocytes, both hypermethylated regions ≥ 80% methylated in both sperm and oocytes, and both hypomethylated regions ≤ 20% methylated in both sperm and oocytes, according to our previously reported data [18]. The proportion of DMVs occupying sperm-specific methylated regions was the highest in the promoter, gene body, CGI, CGI shelf, SINE, LTR, DNA repeat, and simple repeat regions (Fig. 2c and Additional file 10). The proportion of DMVs occupying both hypermethylated regions often followed the proportion of DMVs occupying sperm-specific methylated regions. In addition, these DMVs were incompletely (mosaic) methylated. These data suggested that the sperm-specific methylated regions were more likely to show DMVs just after fertilization in ART. Therefore, we concluded that DMVs might be affected by the techniques of the manipulation used for IVF or ICSI and the culture medium of the fertilized egg.

## Discussion

Using a genome-wide approach, we demonstrated the characteristics of the DNA methylation in ART-SRS patients. They were as follows: (1) the numbers and locations of DMVs were larger and more widespread, respectively. However, some domains such as imprinted regions, SVAs, and retrotransposons had few DMVs. (2) The DMVs tended to more frequently be methylated than demethylated events. (3) The DMVs occurred more frequently in sperm-specific methylated regions. These results may suggest that ART caused the DNA methylation alterations. Major methylation dynamics take place during cell development and the preimplantation stages of embryonic development [19]. ART may prevent the proper erasure, establishment, and maintenance of DNA methylation. Recently, we and others reported that the human paternal genome is globally demethylated after fertilization, which is due to ten–eleven translocation (TET)-mediated active demethylation [18, 20–22]. On the other hand, the maternal genome is demethylated to a much lesser extent in human blastocysts, which was different from the mouse. The pattern of incomplete DMVs we observed in ART-SRS patients might indicate that the changes occurred after fertilization rather than in the gamete. Furthermore, the increased frequency of DMVs in the paternal-specific methylation domain suggested that they occurred just after fertilization.

Previously, we examined the DNA methylation status of 22 germline DMRs (gDMRs) located within the imprinted loci in an ART-BWS and five ART-SRS patients compared to those of Sp-SRS patients [13]. We observed aberrant DNA methylation patterns in the majority of ART patients (1) at multiple imprinted loci, (2) with both maternal and paternal gDMRs, (3) with both hypermethylation and hypomethylation events, and (4) mosaic methylation errors. These aberrant methylation patterns suggested that imprinting defects caused by the global demethylation after fertilization rather than in the gamete might be involved. Others also reported similar results [23, 24]. In this study, we hypothesized that the demethylation and maintenance mechanisms in the preimplantation stage might fail in ART and lead to DMVs and imprinting disorders such as BWS and SRS. Many reports have demonstrated the possibility that the process of ART might affect epigenetic alterations. For example, the phenome of large offspring syndrome has been described in bovine and sheep IVF [25]. Use of sheep embryo cultures in vitro has led to the birth of overweight male offspring with phenotypic similarity to BWS and associated with loss of imprinted methylation at *IGF2R* [26]. Some studies have shown that exposure of mouse embryos to different culture conditions can alter the gene expression and DNA methylation, which could result in abnormal development [27, 28]. Most recent studies have shown an association of human preimplantation embryos after ART with incorrect DNA methylation at gDMRs [29–31]. Surprisingly, day 3 embryos (76%) and blastocysts (50%) after ART had a high frequency of imprinted methylation errors at three gDMRs [31].

In this study, the frequencies of ART-BWS and ART-SRS were still high and the frequency of ART-PWS patients increased more than threefold compared to our 2009 survey [13]. Mussa et al. reported that ART entails a tenfold increased risk of BWS [6]. We reconfirmed that ART increases risk of not only BWS but also SRS and PWS. The frequency of paternal uniparental disomy of chromosome 11 (UPD(11)pat) in Sp-BWS increased approximately two times compared with previous data [32]. Sasaki et al. reported that the frequency of hypomethylation in *KCNQ1OT1*:transcriptional start site (TTS)-DMR in Japanese BWS patients was lower than that in North American/European BWS patients [33]. In addition, we compared the frequency of UPD(11)pat in Sp-BWS patients stratified according to maternal age but there was no correlation between UPD(11)pat and maternal age. Based on the above, the increased frequency of UPD(11)pat in Sp-BWS might be due to race rather than maternal age. Matsubara et al. reported that advanced maternal age affected the onset of PWS through UPD(15)mat, resulting from a disjunction error during meiosis I in oocytes [34]. In stratified analyses of the pathogenesis of PWS according to maternal age, the rate of DNA methylation errors in ART-PWS patients was significantly increased compared with Sp-PWS patients when maternal age was ≤ 37 years, while the rate of UPD(15)mat in ART-PWS patients was significantly increased compared with that in Sp-PWS patients with maternal age ≥ 38 years. These results suggested that the increasing incidence of ART-PWS might be affected not only by maternal age but also DNA methylation altered by ART. The major pathogenesis of AS is deletion or mutation of the *UBA3A* gene on chromosome 15q11.2-q13 and in total the paternal UPD and imprinting defects, which might be associated with maternal age and ART, are less than 5% of the pathogenesis of AS [35]. Therefore, it may not be possible to observe the increased frequency of ART-AS in this study.

We showed that phenotypic differences between ART patients and Sp patients were largely unreported, while changes to phenotypes might be altered by the frequency and the degree of epimutations caused by ART. Lim et al. reported that ART-BWS patients had a significantly lower frequency of exomphalos and higher risk of non-Wilms’ tumor neoplasia [14]. However, we found no major differences in clinical features between the ART-BWS and Sp-BWS patients. The ART-SRS patients having defects at additional loci and DMVs other than at the domain responsible for that disorder displayed additional minor phenotypes, heart malformation, tumorigenesis, diabetes, and mental retardation. For mental retardation, Zhu et al. reported that children born after ART had a slight delay in motor and cognitive development compared with children born to infertile couples who conceived naturally, and it suggested that ART may increase the risk of mental retardation [36]. The higher frequency of mental retardation in ART-SRS patients compared with Sp-SRS patients may be affected by the risk of ART being added to the risk of SRS itself. Since it is unlikely that methylomes in peripheral blood precisely reflect phenotypes of imprinting disorders, we might not be able to clarify the relationship between phenotypes and epimutations. In addition, it may be that DMVs alone have not reached the threshold for the appearance of clinical phenotypes.

Of the 79 genes with DMVs, 19 candidate genes might be involved in clinical symptoms of SRS. *TSPAN1*, *LIMS2*, *SCHIP1*, *HOXA5*, *ABALON*, and *AATBC* genes might be involved in the growth restriction phenotype of SRS. The *SCHIP1* gene is associated with facial size and shape in African children [37] and *Schip1*-knockout mice have skeletal, craniofacial, and digestive problems [38]. *HOXA5* regulates gene expression, morphogenesis and differentiation, and upregulates the tumor suppressor *p53* [39]. *ABALON* and *AATBC* are involved in apoptosis, which plays an important role in embryonic development [40]. *ELEN*-*AS1*, *NPM2*, *GSTO2*, *H19*, *KCNQ1OT1*, *MEG3*, and *BANP* might be involved in tumorigenesis. *H19*, *KCNQ1OT1*, and *MEG3* are also known imprinted genes that function as tumor suppressors. The main clinical phenotype of SRS is growth retardation. A tumor is a type of abnormal and excessive growth of cells. These are opposite growth disturbances. Nevertheless, we observed tumorigenesis in ART-SRS patients in our epidemiological study. In addition, at least three papers have reported tumorigenesis in SRS patients [41–43]. Therefore, we focused on genes related to tumorigenesis. *SLC25A25* and *TCF7L2* might be involved in hypoglycemia. *SLC25A25* encodes an ATP-Mg/Pi inner mitochondrial membrane solute transporter and *Slc25a25*-knockout mice are metabolically inefficient [44]. The Wnt signaling pathway regulator *TCF7L2* is associated with an increased risk of type 2 diabetes and negative regulators of hepatic gluconeogenesis [45]. *RIPOR2* might be involved in defective hearing since it encodes a plasma membrane-associated protein of hair cell stereocilia that is essential for hearing [46]. *CMYA5*, *PRKAG2*, and *CTF1* might be involved in heart malformation. *CMYA5* is associated with left ventricular hypertrophy [47]. *PRKAG2* is associated with glycogen-storage cardiomyopathy [48] and *CTF1* induces cardiac myocyte hypertrophy in vitro [49].

There are some limitations in this study. First, we could not recruit a sufficient number of ART patients with four imprinting disorders to compare with Sp patients for clinical phenotypes and pathogeneses and find any novel appearance for the imprinting disorders in the ART patients. However, there might be differences between the two groups that were not dealt with in our questionnaires. Second, there may be additional strong associations with imprinting disorders and ART because the details about whether or not ART treatments were employed are unknown for half of the patients with imprinting disorders. Third, we cannot provide the reasons for the use of ART for ART-associated patients because we did not inquire about the reason for using ART in the questionnaire. Fourth, we analyzed genome-wide methylation levels in peripheral blood using RRBS technology, which can only cover approximately 10% of genomic CpG cytosines. However, a strength of our study is that the sample group consisted of only Japanese subjects. The Japanese are a relatively homogeneous people geographically and traditionally, because Japan is a small island country and due to the Japanese temperament. Furthermore, genetic differences might affect clinical phenotypes and pathogeneses of imprinting disorders because they are present in the gene-specific DNA methylation levels at birth [50].

## Conclusions

We demonstrated increased frequencies of ART-BWS, ART-SRS, and ART-PWS compared with the live birth rate after ART from 1985 through 2015 in Japan and the characteristics of incomplete and broader DMVs in ART-SRS patients. We reconfirmed the association between ART and three imprinting disorders (BWS, PWS, and SRS). This is perhaps not surprising given the major epigenetic events that take place during early development at a time when the epigenome is most vulnerable. The numbers of children with imprinting disorders born after ART treatment remain limited and the vast majority of children born after such treatments are healthy. On the other hand, it is also well known that epigenetic alterations can increase the risks for various diseases later in life such as cardiovascular disease, type 2 diabetes, and hypertension [51]. Further large-scale, long-term studies will be needed to determine the associations with epimutations and phenotypes of children conceived after ART.

## Materials and methods

### Nationwide search of four congenital imprinting disorders

The protocol was almost the same as that we used previously and was established by the Research Committee on the Epidemiology of Intractable Diseases. The nationwide survey used a two-stage postal method [13]. First, a survey was conducted to estimate the number of individuals with any of the four imprinting diseases: BWS, SRS, PWS, and AS. In the following stage, a second survey was used to identify the clinico-epidemiological features of these syndromes. We previously reported the questionnaires in detail [13].

### Reduced representation bisulfite sequencing

Genomic DNA was obtained from whole blood using the standard extraction protocol and treated with PureLink RNase A (Thermo Fisher Scientific, Waltham, MA, USA). The DNA was used for reduced representation bisulfite sequencing (RRBS) library construction as previously described with modifications [52]. Briefly, 20 ng of genomic DNA was digested with 20 units of *Msp*I enzymes (NEB, Beverly, MA, USA) in 15 μl reactions with 1x NEBuffer 2 at 37 °C for 16 h and 80 °C for 20 min. The digested products were filled in, and an adenosine was added with 10 units of Klenow Fragment (3′ → 5′ *exo*^−^, NEB), 4 μM dGTP, 4 μM dCTP, 40 μM dATP, and 1× NEBuffer 2 to a final volume of 17 μl. The end-repaired/dA-tailing reaction was incubated at 30 °C for 20 min, 37 °C for 20 min, and 75 °C for 20 min. The end-repaired fragments were immediately ligated to 1 μM pre-annealed methylated adaptors using 2000 cohesive end units of T4 DNA Ligase (NEB) in 20 μl reactions with 1× Ligase Reaction Buffer. The ligation reactions were incubated at 16 °C for 16 h and 65 °C for 20 min. The ligated products were separated on 3% NuSieve 3:1 agarose gel (Lonza Japan, Tokyo, Japan) by electrophoresis to isolate 150–350 bp fragments and size-selected fragments were extracted and purified using a MinElute Gel Extraction kit (Qiagen, Valencia, CA, USA) and 20 μl of warmed EB buffer. Then bisulfite conversion was performed using an EpiTect Bisulfite Kit (Qiagen). To incorporate the sample-specific index and amplify the library, PCR was performed with KAPA HiFi HotStart Uracil+ ReadyMix (Kapa Biosystems, Woburn, MA). The PCR amplification was carried out as follows: an initial denaturation at 95 °C for 2 min, 14 cycles of 98 °C for 20 s, 65 °C for 30 s and 72 °C for 30 s, and a final 1-min extension at 72 °C. The RRBS libraries were purified twice using Agencourt AMPure XP beads (Beckman Coulter, Brea, CA, USA), quantified with KAPA Library Quantification Kit (Kapa Biosystems), and sequenced on an HiSeq 2500 platform (Illumina, CA, USA) with 101-bp single-end reads using a TruSeq SR Cluster Kit v3-cBot-HS and TruSeq SBS Kit v3-HS (Illumina). Sequenced reads were processed using an Illumina standard base-calling pipeline (v1.8.2) and the index and adapter sequences were removed. After trimming the first and last four bases, the reads were aligned to Human Genome assembly (GrCh37/hg19) using Bismark (v.0.9.0) with default parameters [53]. Reads that showed < 90% bisulfite conversion were filtered to remove those that resulted from incomplete bisulfite-converted molecules. The methylation level of each cytosine was calculated using the Bismark methylation extractor. We analyzed only CpG cytosines covered with ≥ 5 reads.

### Annotations of genomic regions

Annotations of Refseq genes and repeat sequences were downloaded from the UCSC Genome Browser (https://genome.ucsc.edu/). Refseq genes shorter than 300 bp (encoding microRNAs or small nucleolar RNAs) were excluded from our analyses. Promoters were defined as regions 500 bp upstream and downstream from the transcription start sites of the Refseq transcripts. For calculation of the mean methylation levels, we analyzed the genomic regions (promoters, exons, introns, intergenic regions, imprinted gDMRs, CGIs, CGI shores, and CGI shelves) containing ≥ 10 CpG cytosines and SINEs, LINEs, LTRs, DNA repeats, SVAs, and simple repeats containing ≥ 3 CpG cytosines with sufficient coverage for calculation of the methylation levels. Regions and names of the 21 imprinted gDMRs were defined as previously reported [54].

### Statistical analysis

Statistical analyses were performed using R or JMP Pro 13.1.0 (SAS Institute, Cary, NC, USA). Statistical significance among the clinical characteristics was determined using Fisher’s exact test or the Mann–Whitney *U* test at *p* < 0.05. Correlations of all CpG cytosines among individual ART-SRS patients, Sp-SRS patients, and ten spontaneously conceived normal children (control) were calculated using the Pearson correlation coefficient. Statistical significance in the mean methylation levels of various genomic regions among the three groups was determined using Steel’s test at *p* < 0.05. Using the Mann–Whitney *U* test with the Benjamini-Hochberg correction, we selected the DNA methylation variations (DMVs) showing absolute methylation changes ≥ 7.5% and a FDR < 0.05. Fisher’s exact test was used to calculate the *p* values for the number of DMVs. Fisher’s exact test with the Benjamini-Hochberg procedure was used to calculate the *p* values for enriched GO terms in DAVID analysis [55].

## Additional files

Additional file 1: The numbers and age distributions of patients with the four imprinting diseases. (a) BWS. (b) AS. (c) PWS. (d) SRS. The vertical axis shows the number of patients and the horizontal axis shows age. (JPG 1026 kb)

Additional file 2: Flowchart showing the molecular testing in four imprinted disorders. (a) BWS. (b) AS. (c) PWS. (d) SRS. The new BWS consensus score [32] and the Netchine-Harbison clinical scoring system (NH-CSS) were used for the diagnoses of BWS and SRS, respectively. NH-CSS, Netchine-Harbison clinical scoring system; UPD, uniparental disomy; GOM, gain of methylation; LOM, loss of methylation. (JPG 1285 kb)

Additional file 3: Clinical characterization of ART-patients. OI/TI, ovulation induction/timed intercourse; IUI, intrauterine insemination; IVF, in vitro fertilization; ICSI, intracytoplasmic sperm injection. N.A. indicates that data was not available. Clinical features were as follows, 1. Macroglossia, 2. Earlobe creases, 3. Umbilical hernia, 4. Hemihypertrophy, 5. Exomphalos, 6. Exophthalmos, 7. Hepatomegaly, 8. Nephromegaly, 9. Ocular hypertelorism, 10. Cryptorchism, 11. Occlusal interference, 12. Mental retardation, 13. Epilepsy, 14. Dysphasia, 15. Dyschromatosis, 16. Ictal laughter, 17. Prognathism, 18. Hyposomnia, 19. Convulsions, 20. Microcephaly, 21. Hypotonia, 22. Feeding difficulties, 23. Almond-shaped eyes, 24. Short stature, 25. Triangular mouse, 26. Acromicria, 27. Bulimia, 28. Obesity, 29. Hypogonadism, 30. Diabetes, 31. Gastrointestinal injury, 32. Cardiac failure, 33. Failure to thrive, 34. Triangular-shaped face, 35. Body asymmetry, 36. Clinodactyly of the fifth fingers, 37. Sweating, 38. Heart malformation. 39. Tumorigenesis. (XLSX 12 kb)

Additional file 4: Frequency of different prevalence of UPD(15)mat and DNA methylation error in ART-patients and Sp-patients with PWS according to maternal age. All data were obtained from the questionnaire. DNA methylation error indicates gain of methylation at *SNRPN*-DMR. UPD(15)mat, maternal uniparental disomy of chromosome 15. (XLSX 11 kb)

Additional file 5: Frequency of different pathogeneses in ART-patients and Sp-patients with BWS, AS and SRS stratified according to maternal age. (a) BWS. (b) AS. (c) SRS. The numbers and percentages of patients with chromosomal abnormalities, gene mutations and methylation abnormalities were obtained from a questionnaire. For BWS, UPD and gene indicate paternally uniparental disomy of chromosome 11 and *CDKN1C*, and methylation errors include both gain of methylation at *H19/IGF2* IG-DMR and loss of methylation (LOM) at *KCNQ1OT1*:TSS-DMR, respectively. For AS, UPD and gene indicate paternally uniparental disomy of chromosome 15 and *UBE3A*, respectively. For SRS, UPD and methylation error indicate maternally uniparental disomy of chromosome 7 and LOM at *H19/IGF2* IG-DMR, respectively. UPD, uniparental disomy; LOM, loss of methylation. (XLSX 11 kb)

Additional file 6: Sequencing information. (XLSX 10 kb)

Additional file 7: Correlations of the methylation levels of all CpG cytosines covered in all samples. (JPG 832 kb)

Additional file 8: Methylation levels of various genomic features in autosomes. Mean methylation levels (%) of CpG cytosines in the promoter, gene body, exon, intron, intergenic region, CGI, CGI shore, CGI shelf, SINE, LINE, LTR, DNA repeat element, SVA and simple repeat. Data are shown as mean ± standard deviation. Since the numbers of ART-SRS and Sp-SRS groups were small, we did not calculate *p*-values between two groups. SINE, short interspersed nuclear element; LINE, long interspersed nuclear element; LTR, long terminal repeat element; SVA, SINE-VNTR-Alu. (XLSX 11 kb)

Additional file 9: Lists of DMVs in various genomic regions. N.A. indicates that data was not available. (XLSX 1005 kb)

Additional file 10: DMVs in ART-SRS and Sp-SRS patients classified based on methylation of gametes. Sperm-specific methylated regions were ≥ 80% methylated in sperm and ≤ 20% methylated in oocytes, oocyte-specific methylated regions were ≤ 20% methylated in sperm and ≥ 80% methylated in oocytes, both hypermethylated regions were ≥ 80% methylated in both sperm and oocytes, both hypomethylated regions were ≤ 20% methylated in both sperm and oocytes according to our previously reported data [18]. N.A. indicates that data was not available. (JPG 1269 kb)

## Abbreviations

ART: Assisted reproductive technologies
AS: Angelman syndrome
ATP: Adenosine triphosphate
BWS: Beckwith-Wiedemann syndrome
CGI: CpG island
DAVID: Database for Annotation, Visualization and Integrated Discovery
DDBJ: DNA Data Bank of Japan
DMR: Differentially methylated region
DMV: DNA methylation variation
FDR: False discovery rate
gDMR: Germline differentially methylated region
GO: Gene ontology
ICSI: Intracytoplasmic sperm injection
IG: Intergenic
IVF: In vitro fertilization
LINE: Long interspersed nuclear element
LTR: Long terminal repeat
N.A.: Not available
PWS: Prader-Willi syndrome
RRBS: Reduced representation bisulfite sequencing
SINE: Short interspersed nuclear element
Sp: Spontaneously conceived
SRS: Silver-Russell syndrome
SVA: SINE-VNTR-Alu
TET: Ten–eleven translocation
TSS: Transcriptional start site
UPD: Uniparental disomy
UPD(11)pat: Paternal uniparental disomy of chromosome 11
UPD(15)mat: Maternal uniparental disomy of chromosome 15
VNTR: Variable number of tandem repeat
