# Supplementary figures and images for: Association of four imprinting disorders and ART
Source: Clin Epigenetics. 2019 Feb 7;11:21. doi: 10.1186/s13148-019-0623-3 (PMC6367766; doi:10.1186/s13148-019-0623-3)

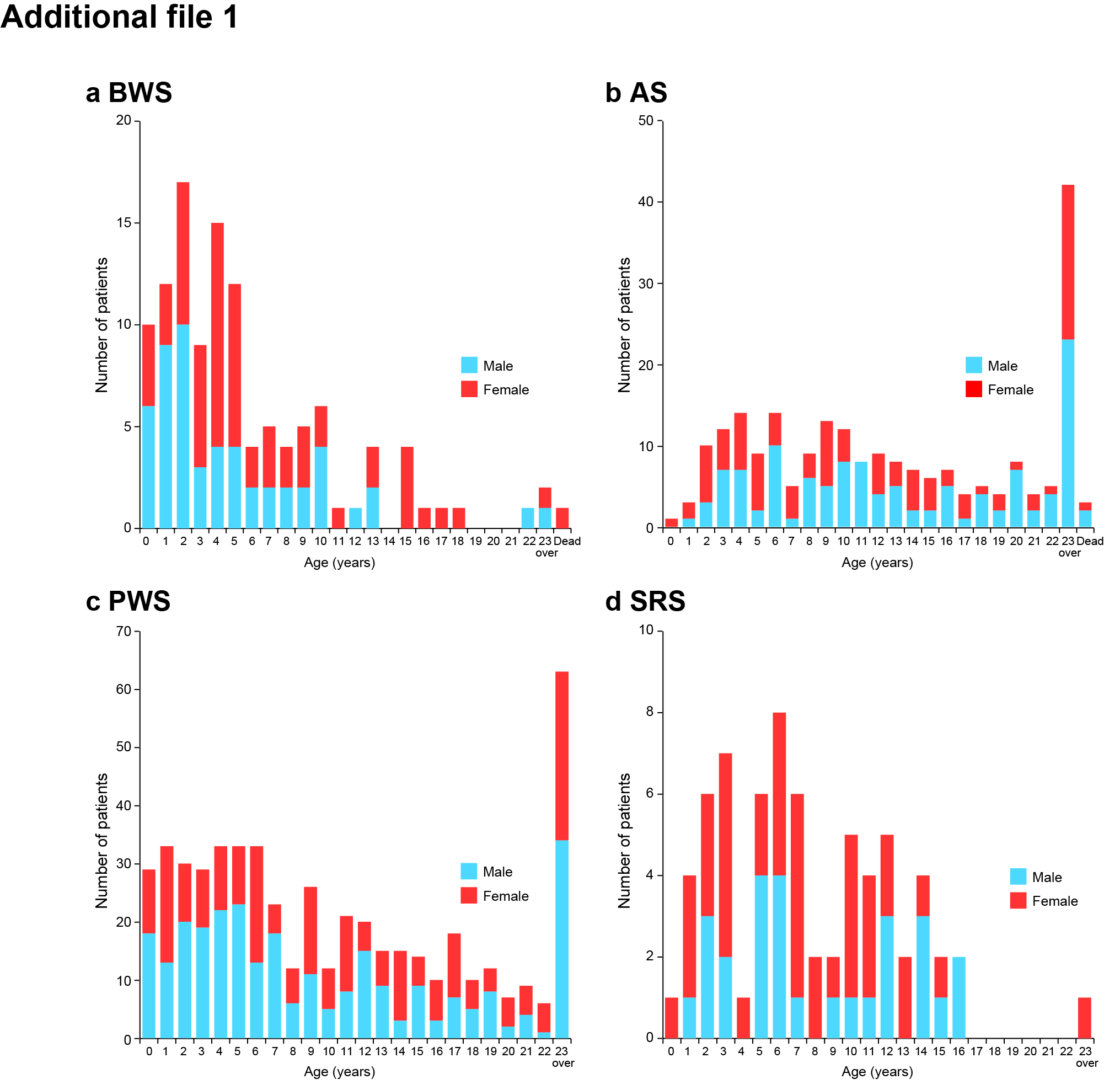

Supplement: Supplementary file 1 — The numbers and age distributions of patients with the four imprinting diseases. (a) BWS. (b) AS. (c) PWS. (d) SRS. The vertical axis shows the number of patients and the horizontal axis shows age. (JPG 1026 kb) [file 13148_2019_623_MOESM1_ESM.jpg]

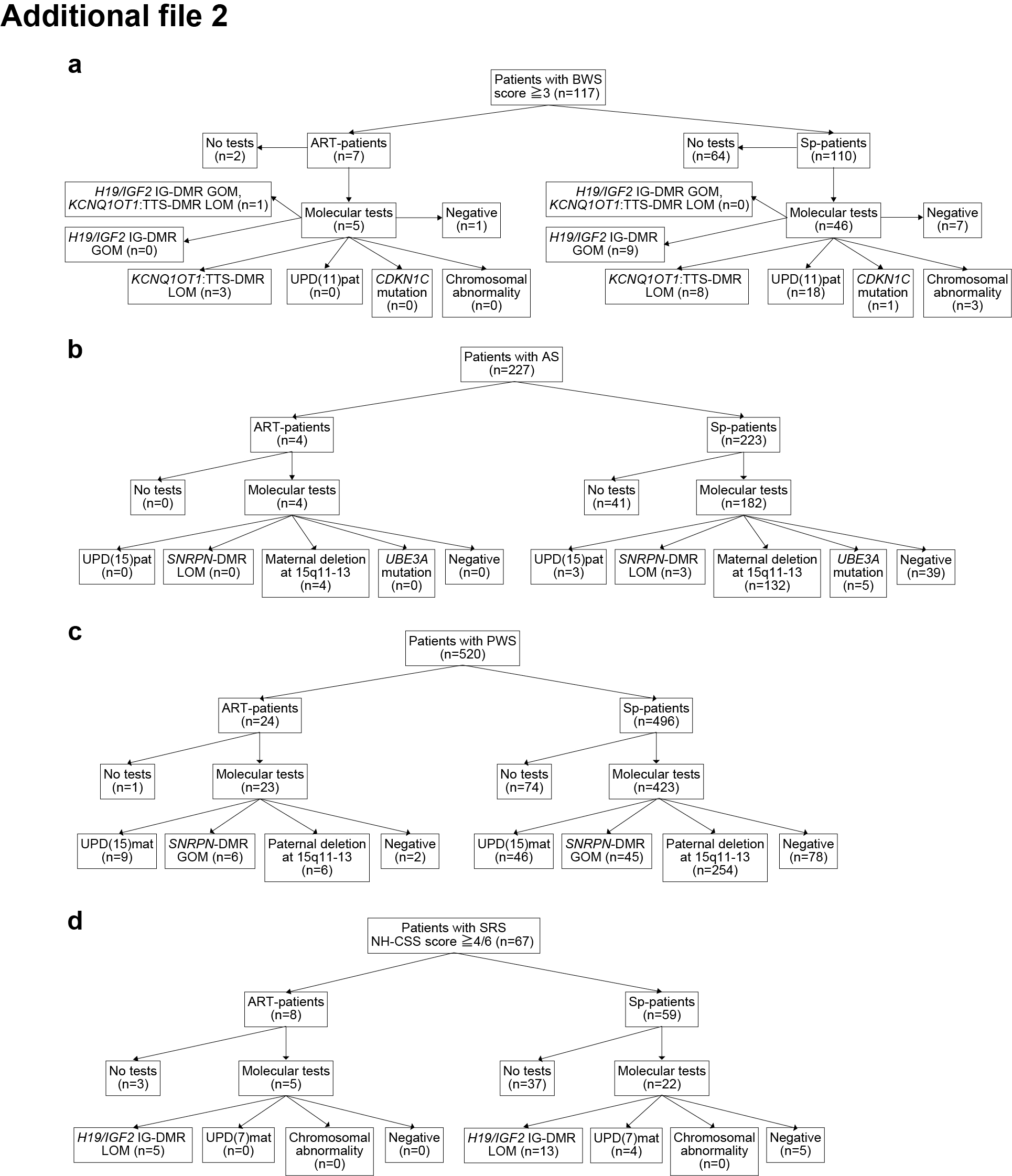

Supplement: Supplementary file 2 — Flowchart showing the molecular testing in four imprinted disorders. (a) BWS. (b) AS. (c) PWS. (d) SRS. The new BWS consensus score [32] and the Netchine-Harbison clinical scoring system (NH-CSS) were used for the diagnoses of BWS and SRS, respectively. NH-CSS, Netchine-Harbison clinical scoring system; UPD, uniparental disomy; GOM, gain of methylation; LOM, loss of methylation. (JPG 1285 kb) [file 13148_2019_623_MOESM2_ESM.jpg]

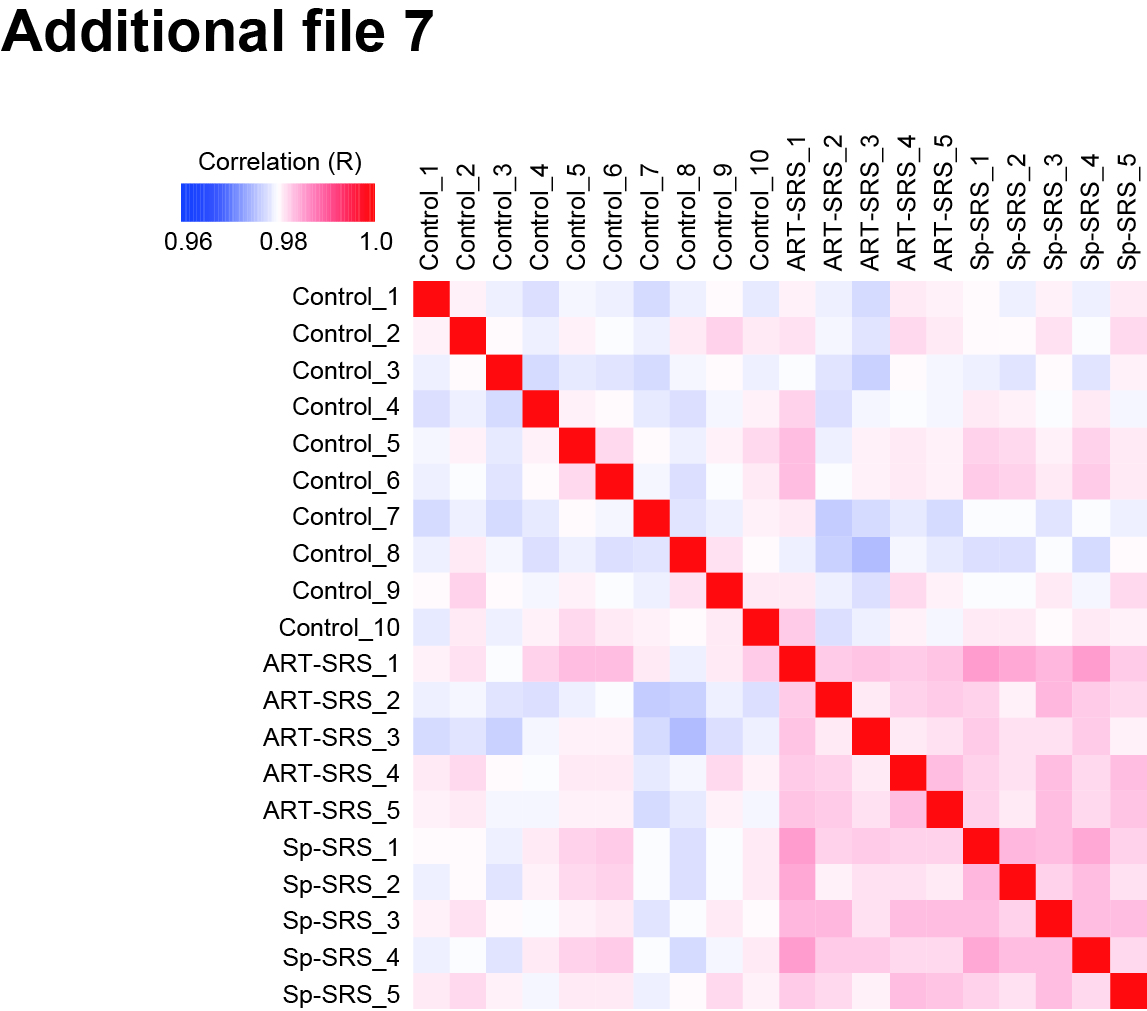

Supplement: Supplementary file 7 — Correlations of the methylation levels of all CpG cytosines covered in all samples. (JPG 832 kb) [file 13148_2019_623_MOESM7_ESM.jpg]

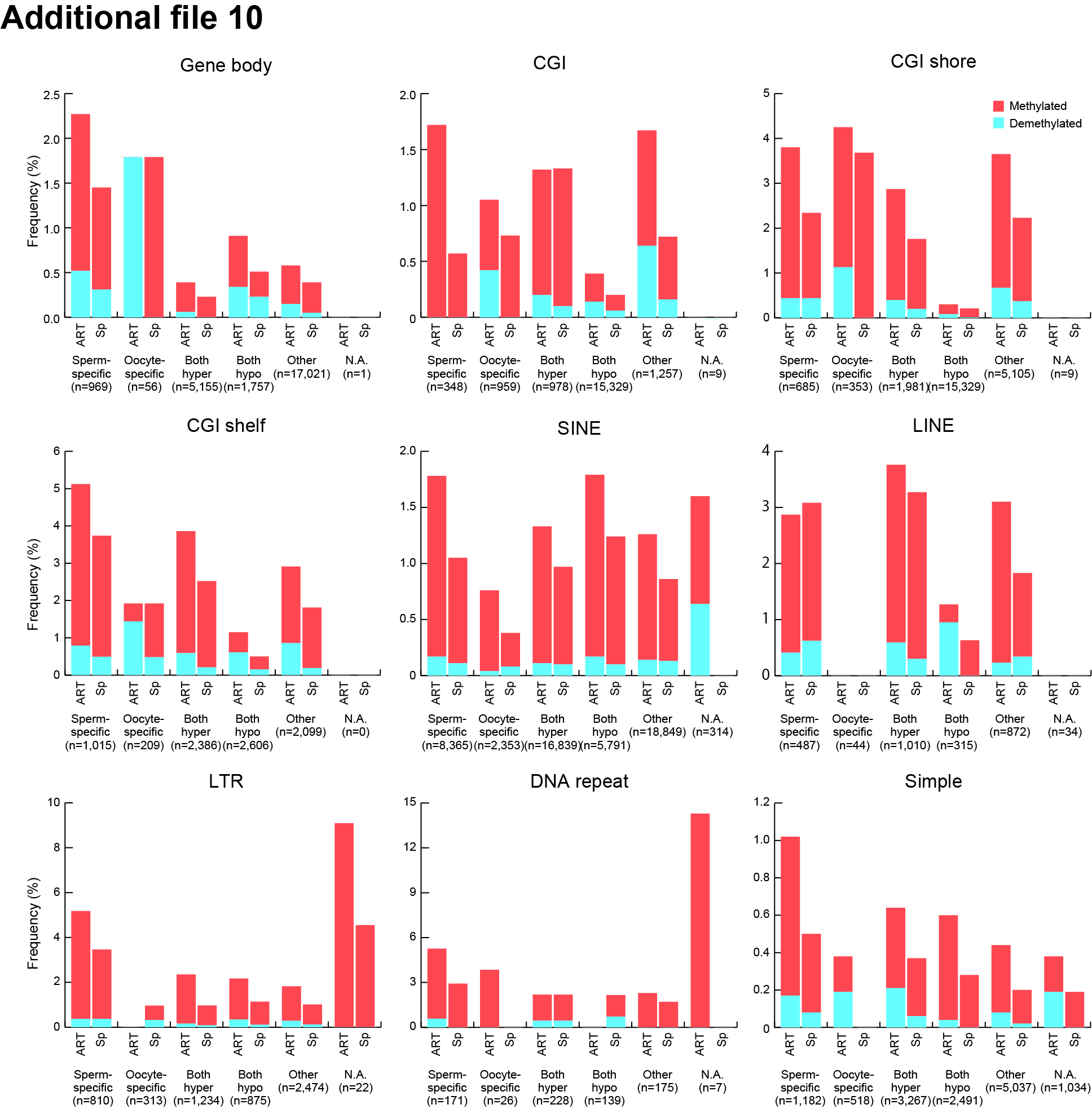

Supplement: Supplementary file 10 — DMVs in ART-SRS and Sp-SRS patients classified based on methylation of gametes. Sperm-specific methylated regions were ≥ 80% methylated in sperm and ≤ 20% methylated in oocytes, oocyte-specific methylated regions were ≤ 20% methylated in sperm and ≥ 80% methylated in oocytes, both hypermethylated regions were ≥ 80% methylated in both sperm and oocytes, both hypomethylated regions were ≤ 20% methylated in both sperm and oocytes according to our previously reported data [18]. N.A. indicates that data was not available. (JPG 1269 kb) [file 13148_2019_623_MOESM10_ESM.jpg]
